# SARS-CoV-2 induced thrombocytopenia as an important biomarker significantly correlated with abnormal coagulation function, increased intravascular blood clot risk and mortality in COVID-19 patients

**DOI:** 10.1186/s40164-020-00172-4

**Published:** 2020-07-17

**Authors:** Changqian Bao, Xiandong Tao, Wei Cui, Bin Yi, Tiewen Pan, Ken H. Young, Wenbin Qian

**Affiliations:** 1 Department of Hematology, The Second Affiliated Hospital, Zhejiang University School of Medicine, Hangzhou, 310009 China; 2 Program in Clinical Medicine, Zhejiang University School of Medicine, Hangzhou, 310058 China; 3 Wuhan Huoshenshan Hospital, Wuhan, 430100 China; 4 The Third Affiliated Hospital, Naval Medical University of Shanghai, Shanghai, 200438 China; 5 Department of Intensive Care Unit, the Second Affiliated Hospital, Zhejiang University School of Medicine, Hangzhou, 310009 China; 6 Hematopathology Division and Department of Pathology, Duke University Cancer Center, Durham, NC USA

**Keywords:** COVID-19, SARS-CoV-2, Thrombocytopenia, Coagulation, DIC

## Abstract

**Background:**

Coronavirus disease 2019 (COVID-19) is a novel infectious viral disease, which lacks well-established diagnostic laboratory parameters that could be used to evaluate disease severity, thromboembolism or cardiovascular events and to predict clinical prognosis. Coagulation cascade and platelet functions have not been well studied in the COVID-19 patients.

**Methods:**

A total of 178 patients enrolled in Wuhan Huoshenshan Hospital were included for the study. Blood platelets and coagulation functions were analyzed in COVID-19 patients with non-severe and severe subgroups. Other biochemical laboratory parameters were also analyzed.

**Results:**

Forty-nine (27.5%) out of 178 patients were diagnosed with severe disease in this study, and 129 patients with non-severe disease. Severe disease group had significant lower platelet count 186.00 (103.50–249.00) ×10^9^/L than 251.00 (202.00–317.00) ×10^9^/L of non-severe group, *p *= 0.000. Severe group also had significantly abnormal coagulation parameters than non-severe group: prothrombin time (PT) 14.55 (13.40–16.53) s vs. 12.70 (12.15–13.59) s, *p *= 0.000; international normalized ratio (INR) 1.21 (1.13–1.36) vs. 1.06 (1.01–1.13), *p *= 0.000; thrombin time (TT) 16.35 (15.69–17.47) s vs. 15.68 (14.79–16.69) s, *p *= 0.011; D-Dimer 1.05 (0.68–5.90) mg/L vs. 0.42 (0.28–0.79) mg/L, *p *= 0.000; While the liver function parameter alanine aminotransferase (ALT) and aspartate aminotransferase (AST) didn’t show significance between two groups, ALT 30.80 (19.00–58.30) IU/L vs. 28.80 (15.75–50.15) IU/L, *p *= 0.487; AST 27.80 (19.30–40.55) IU/L vs. 22.6 (16.7–32.03) IU/L, *p *= 0.102. Disseminated intravascular coagulation (DIC) rate was 6.1% in severe group while 0% in non-severe group. Survival rate of severe disease group was worse than non-severe group, 85.7% vs. 100%, *p *= 0.000. Thrombocytopenia correlated with coagulation function, DIC rate and survival. Six out of 7 death case had thrombocytopenia during hospitalization, and platelet count decreased subsequently until death. Thrombocytopenia occurred within 1 week after admission in 6 recovered patients. And increased platelet levels followed by positive SARS-CoV-2 IgM/IgG and negative coronavirus nucleic acid tested in 8 recovered patients.

**Conclusions:**

Low platelet count is associated with abnormal coagulation function and increased risk of DIC, severe disease manifestation and increased mortality in patients with COVID-19.

## Background

A novel coronavirus, severe acute respiratory syndrome coronavirus 2 (SARS-CoV-2), has spread globally since December 2019. Common symptoms for coronavirus disease 2019 (COVID-19) includes fever, cough, acute respiratory distress syndrome, and cardiovascular events. Hematopoiesis could also be affected by infection of SARS-CoV-2. However, thrombocytopenia, abnormal coagulation function and risk of disseminated intravascular coagulation (DIC) haven’t been well analyzed in COVID-19 patients yet. A national study in China reported platelet count less than 150 ×10^9^/L happened with 36.2% in all COVID-19 patients [1], 31.6% in non-severe and 57.7% in severe patients, while another study showed the frequency of thrombocytopenia was 12% [2]. In this study, we focus on the event of thrombocytopenia, abnormal coagulation function and DIC, and how these dysregulated abnormalities could impact the outcome and the mortality of patients.

## Methods

A total of 178 hospitalized patients in Huoshenshan hospital between Feb 10, 2020 and March 15, 2020 was prospectively enrolled, followed and analyzed. The study was approved by ethics committee and institution research board of Huoshenshan hospital and the Second Affiliated Hospital, Zhejiang University School of Medicine. Written informed consent was waived due to urgent need of data collection. Patients tested positive by real-time reverse transcription PCR (real-time RT-PCR) and diagnosed according to WHO interim guidance [3] were enrolled in this study.

All patients had required laboratory testing and chest computerized tomography (CT). All laboratory tests were performed in compliance with the clinical needs of patients. Detailed clinical symptom presentations, laboratory findings including blood routine count, coagulation function, blood biochemistry, SARS-CoV-2 nucleic acid and antibody test, and radiographic evaluation were dynamically analyzed. Blood draws were taken at least once a week since admission for blood routine and other biochemistry tests if needed. We defined the severity of disease as non-severe and severe subtype when patients were admitted according to American Thoracic Society guidelines for community-acquired pneumonia by clinicians [4]. The guideline includes either one major criteria or three or more minor criteria. Minor criteria: respiratory rate more than or equal to 30 per minute, Pa_O2_/Fi_O2_ less than or equal to 250, multilobar infiltrates, confusion or disorientation, blood urea nitrogen level more than or equal to 20 mg/dL, white blood cell less than 4 ×10^9^/L, platelet count less than 100 ×10^9^/L, hypothermia (core temperature less than 36 °C), hypotension requiring aggressive fluid resuscitation; Major criteria: septic shock with need for vasopressors, respiratory failure requiring mechanical ventilation.

We analyzed the disseminated intravascular coagulation based on recommended ISTH criteria: platelet counts 50–100 ×10^9^/L as 1 point, less than 50 ×10^9^/L as 2 points; D-dimer 1–3 μg/mL as 2 points, more than 3.0 μg/mL as 3 points; fibrinogen less than 1.0 g/L as 1 point; Prolongation of PT 3–6 s as 1 point, more than 6 s as 2 points; If total points add to more than or equal to 5, it meets the criteria of DIC. Continuous variables were described as median and interquartile range (IQR), while comparison of non-severe and severe subgroups performed by *t* test. Correlation between thrombocytopenia, coagulation functions, DIC rate and survival rate was analyzed by spearman correlation. Statistical analysis was performed with the Social Sciences (SPSS) version 22.0 and GraphPad Prism 8.

## Results

178 patients diagnosed with in SARS-CoV-2 enrolled were enrolled in this study, and all the patients were confirmed by real-time PCR. Seventy-two (40.4%) patients were female, and 106 (59.6%) patients were male. The median age of all patients was 64 years old, and only 16.9% patients had fever when admitted into hospital. Ninety-three patients have coexisting disorders, including hypertension (32.6%), diabetes (17.4%), coronary heart disease (5.6%), hepatitis B infection (3.4%), chronic obstructive pulmonary disease (6.2%), cerebrovascular disease (1.7%), chronic renal disease (1.7%) and cancer (1.1%). Most patients were non-severe patients (72.5%), while 27.5% were severe patients (Table 1).

**Table 1:** Clinical characteristics of patients

All patient (N = 178)	N	%
Gender		
Female	72	40.4
Male	106	59.6
Age (IQR), year	64 (54–70)	
Temperature on admission		
< 37.5 °C	148	83.1
≥ 37.5 °C	30	16.9
Coexisting disorder-no. (%)	93	52.2
Hypertension	58	32.6
Diabetes	31	17.4
Coronary heart disease	10	5.6
Hepatitis B infection	6	3.4
Chronic obstructive pulmonary disease	11	6.2
Cerebrovascular disease	3	1.7
Cancer	2	1.1
Chronic renal disease	3	1.7
Disease severity		
Non-severe	129	72.5
Severe	49	27.5
Outcome		
Death	7	3.93
Survivor	171	96.1

Data are shown as n (%)

Severe patients had higher white blood cell counts than non-severe patients, 7.90 (5.65-10.05) ×10^9^/L vs. 5.40 (4.40–7.05) ×10^9^/L, *p *= 0.000. In our study, severe patients had significantly lower lymphocytes. Severe patients also had higher neutrophils and basophils than non-severe patients, while monocytes, eosinophils and hemoglobin did not show statistical difference (Table 2).

**Table 2:** Laboratory examination at admission

	All patients (N = 178)	Nonsevere (N = 129)	Severe (N = 49)	*P* value
Blood cell counts				
White blood cells (3.5–9.5*10^9^/L)	5.95 (4.50–8.09)	5.40 (4.40–7.05)	7.90 (5.65–10.05)	0.000
Neutrophils (1.8–6.3*10^9^/L)	3.90 (2.66–5.64)	3.51 (2.45–4.85)	6.20 (3.88–8.51)	0.000
Lymphocytes (1.1–3.2*10^9^/L)	1.20 (0.82–1.61)	1.29 (0.98–1.69)	0.73 (0.45–1.22)	0.000
Monocytes (0.1–0.6*10^9^/L)	0.42 (0.30–0.60)	0.42 (0.32–0.62)	0.38 (0.27–0.55)	0.547
Eosinophils (0.02–0.52*10^9^/L)	0.08 (0.03–0.14)	0.09 (0.05–0.14)	0.03 (0.01–0.10)	0.343
Basophils (0–0.06*10^9^/L)	0.02 (0.01–0.03)	0.02 (0.01–0.03)	0.01 (0.00–0.02)	0.004
Hemoglobin (130–175 g/L)	126.00 (115.00–133.25)	126.00 (117.50–134.00)	127.00 (111.50–132.50)	0.114
Platelets (125–350*10^9^/L)	236.00 (169.50–305.25)	251.00 (202.00–317.00)	186.00 (103.50– 249.00)	0.000
Coagulation function				
PT (9.2–15 s)	13.17 (12.30–14.22)	12.70 (12.15–13.59)	14.55 (13.40–16.53)	0.000
INR (0.8–1.25)	1.1 (1.03–1.19)	1.06 (1.01–1.13)	1.21 (1.13–1.36)	0.000
PT % (70–125)	94.3% (89.53%–98.48%)	96.4% (92.1%–99.3%)	87.60% (80.10%–92.15%)	0.000
APTT (21–37 s)	27.54 (25.31–30.18)	25.99 (25.11–29.33)	29.25 (26.95–32.84)	0.057
FIB (2–4 g/L)	3.26 (2.79–3.99)	3.23 (2.80–3.70)	3.62 (2.70–4.33)	0.780
TT (10–20 s)	15.91 (14.94–15.83)	15.68 (14.79–16.69)	16.35 (15.69–17.47)	0.011
D-Dimer (0–0.55 mg/L)	0.56 (0.33–1.12)	0.42 (0.28–0.79)	1.05 (0.68–5.90)	0.000
Blood biochemistry				
ALT (9–50 IU/L)	29.85 (16.60–52.63)	28.80 (15.75–50.15)	30.80 (19.00–58.30)	0.487
AST (9–60 IU/L)	23.90 (17.20–36.60)	22.6 (16.7–32.03)	27.80 (19.30–40.55)	0.102
TP (68–85 g/L)	62.60 (57.50–67.30)	63.95 (67.95– 58.85)	57.80 (52.80–63.15)	0.000
ALB (40–50 g/L)	34.50 (30.30–38.00)	36.00 (32.88–39.10)	30.90 (27.60–33.65)	0.000
GLB (20–35 g/L)	34.50 (30.30–38.00)	26.70 (25.20–29.60)	26.50 (23.90–31.40)	0.576
Blood glucose (3.9–6.11 mmol/L)	5.22 (4.64–6.55)	5.06 (4.61–5.84)	6.15 (5.08–9.16)	0.002
B–type natriuretic peptide (0–100 pg/mL)	11.05 (0.01–48.24)	0.01 (0.01–33.74)	40.55 (2.52–116.64)	0.052
Serum creatinine (57–97 μmol/L)	66.70 (56.83–78.85)	67.35 (57.88–77.75)	65.35 (55.78–79.45)	0.402
LDH(120–250 IU/L)	196.10 (164.80–278.70)	188.25 (162.13–219.33)	302.70 (198.90–479.00)	0.000
Myoglobin (0–65 ng/mL)	7.58 (3.36–21.46)	5.13 (2.92–11.97)	25.14 (9.05–30.22)	0.022
Troponin (0–0.04 ng/mL)	0.01 (0.01–0.01)	0.01 (0.01–0.01)	0.01 (0.01–0.05)	0.125
Infection related parameters				
PCT (0–0.05 ng/mL)	0.05 (0.03–0.12)	0.03 (0.03–0.07)	0.13 (0.07–0.21)	0.564
CRP (0–4 mg/L)	7.25 (1.59–60.74)	4.22 (0.96–15.75)	64.74 (6.58–119.03)	0.000

Data are median value (interquartile range)

*PT* prothrombin time, *INR* international normalized ratio, *APTT* activated partial thromboplastin time, *FIB* fibrinogen, *TT* thrombin time, *LDH* lactate dehydrogenase, *ALT* alanine aminotransferase, *AST* aspartate aminotransferase, *TP* total protein, *ALB* albumin, *GLB* globulin, *PCT* procalcitonin, *CRP* C-reactive protein

At admission, the platelet count was vastly lower in severe patients 186.00 (103.50– 249.00) ×10^9^/L than non-severe patients 251.00 (202.00–317.00), *p *= 0.000, data is shown as median value (interquartile range). Severe group also had significantly abnormal coagulation parameters than non-severe group with prothrombin time (PT) 14.55 (13.40–16.53) s vs. 12.70 (12.15–13.59) s, *p *= 0.000; international normalized ratio (INR) 1.21 (1.13–1.36) vs. 1.06 (1.01–1.13), *p *= 0.000; thrombin time (TT) 16.35 (15.69–17.47) s vs. 15.68 (14.79–16.69) s, *p *= 0.011; D-Dimer 1.05 (0.68–5.90) mg/L vs. 0.42 (0.28–0.79) mg/L, *p *= 0.000;

While the liver function parameter alanine aminotransferase (ALT) and aspartate aminotransferase (AST) didn’t show significance between two subgroups, ALT 30.80 (19.00–58.30) IU/L vs. 28.80 (15.75–50.15) IU/L, *p *= 0.487; AST 27.80 (19.30–40.55) IU/L vs. 22.6 (16.7–32.03) IU/L, *p *= 0.102. Total protein level, albumin (ALB) and blood glucose level revealed significant difference between two groups, whereas Globulin (GLB), B-type natriuretic peptide and serum creatinine did not. The severe patients had significantly higher lactate dehydrogenase (LDH) and C-reactive protein (CRP) level as well.

As we analyzed the disseminated intravascular coagulation based on recommended ISTH criteria, DIC rate was 6.1% in severe group while not seen (0%) in non-severe group (Table 3).

**Table 3:** DIC score of patients at admission

	Score	Non-severe (N = 129)	Severe (N = 49)
Platelet counts (×10^9^/L)			
> 100	0	125 (96.9%)	38 (77.6%)
< 100	1	4 (3.1%)	11 (22.4%)
< 50	2	0 (0%)	0 (0%)
Prolonged PT (s)			
< 3	0	128 (99.2%)	44 (89.8%)
> 3, but < 6	1	0 (0%)	3 (6.1%)
> 6	2	1 (0.78%)	2(4.1%)
D-Dimer (μg/mL)			
< 1	0	95 (73.6%)	21 (42.9%)
> 1, but < 3	2	14 (10.9%)	9 (18.4%)
> 3	3	6 (4.7%)	16 (32.7%)
Fibrinogen level (g/L)			
> 1	0	129 (100%)	49 (100%)
< 1	1	0 (0%)	0 (0%)
ISTH criteria of DIC			
≥ 5		0 (0%)	2 (6.1%)
< 5		129 (100%)	46 (93.9%)

In the spearman correlation analysis (Table 4), thrombocytopenia at admission had significant correlation with coagulation parameters PT (*p *= 0.000), APTT (*p *= 0.016), and level of D-Dimer (*p *= 0.000), Thrombocytopenia at admission also has significant correlation with DIC rate (*p *= 0.000), but not with survival rate (*p *= 0.345). While thrombocytopenia at 1 week after admission had significant correlation with survival (*p *= 0.019).

**Table 4:** Correlation of Thrombocytopenia, coagulation functions, DIC rate and survival rate

Thrombocytopenia	PT	APTT	Fib	TT	D-Dimer	DIC	Disease severity	Death
At admission								
Correlation coefficient	0.265**	0.180*	− 0.104	0.067	0.310**	0.311**	0.351**	0.071
Significance	0.000	0.016	0.167	0.378	0.000	0.000	0.000	0.347
One week after admission								
Correlation coefficient								0.176*
Significance								0.019

Data are shown as n (%)

**Fig. 1:**
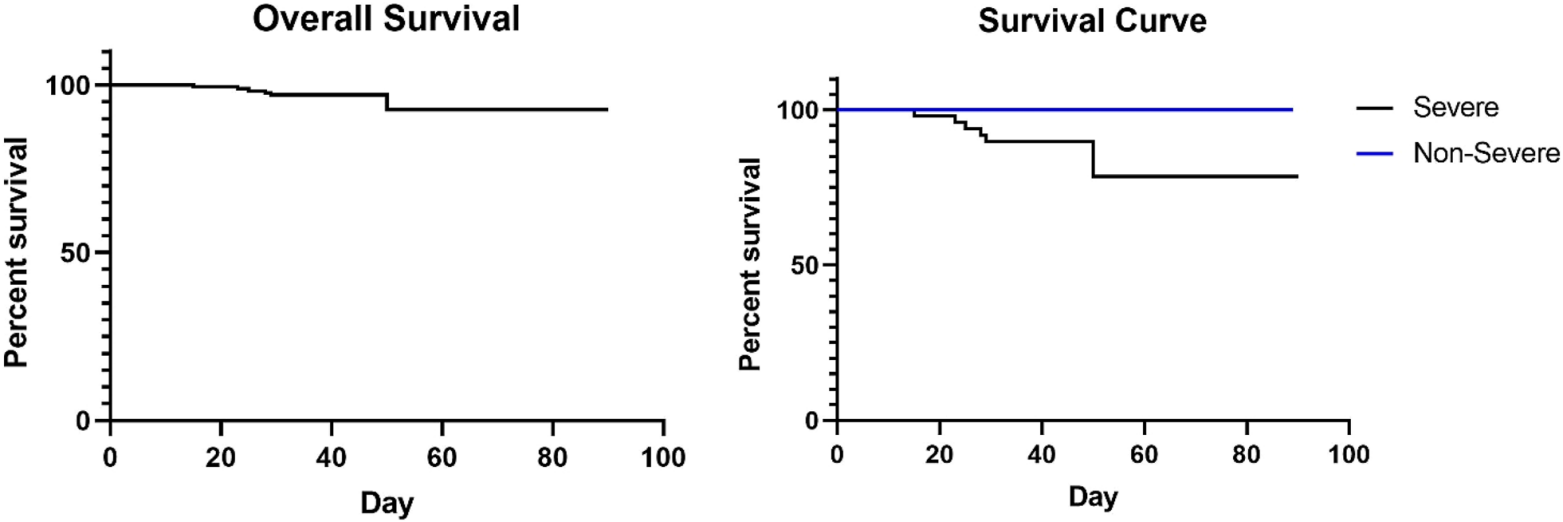
Survival curve

**Fig. 2:**
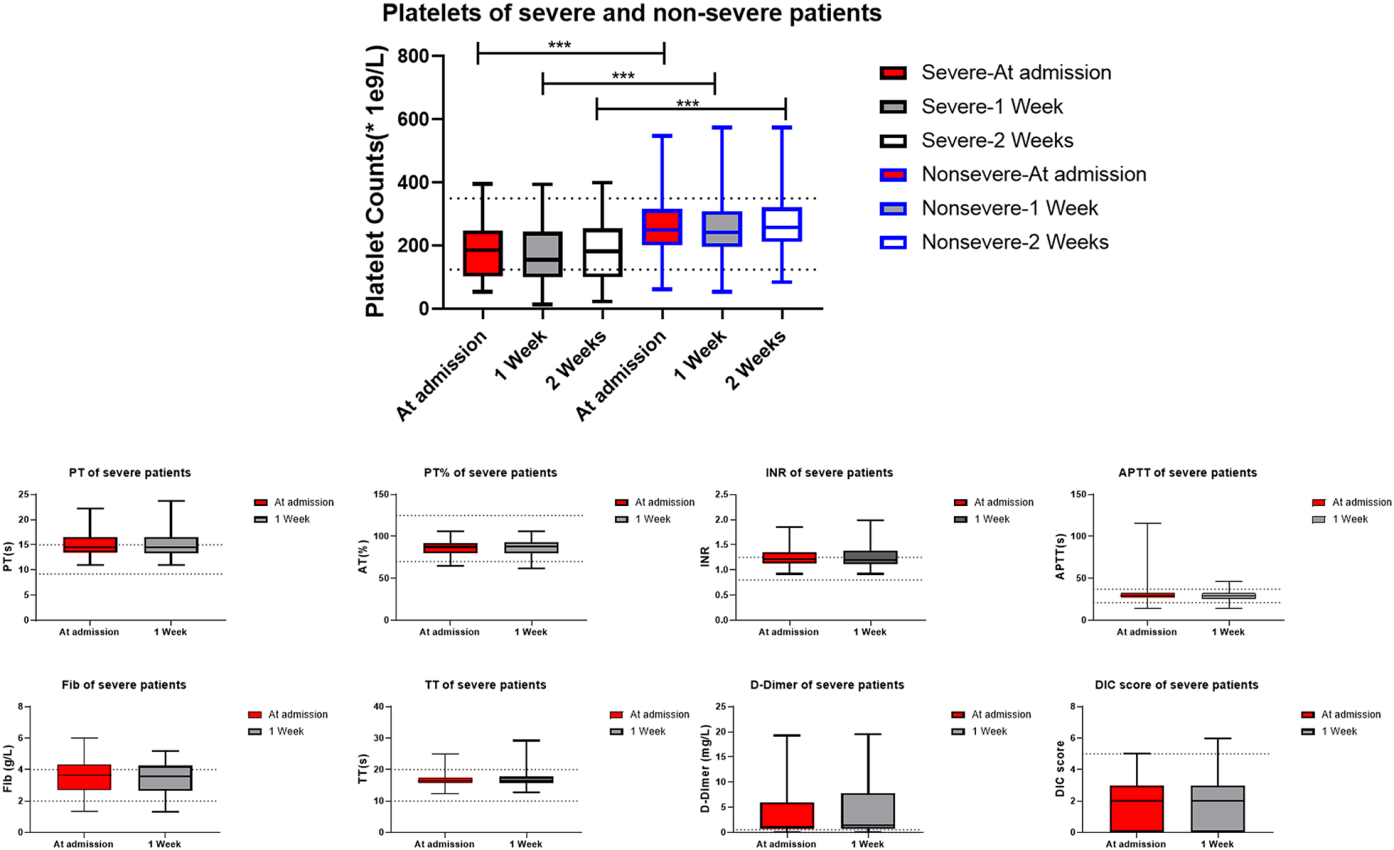
Coagulation function parameters of patients. Each box extends from the 25th to 75th percentiles, and each whisker goes down to the smallest value and up to the largest value, ***p < 0.001. Death cases 1, 2, 3, 4 didn’t have data at 2 weeks after admission

**Fig. 3:**
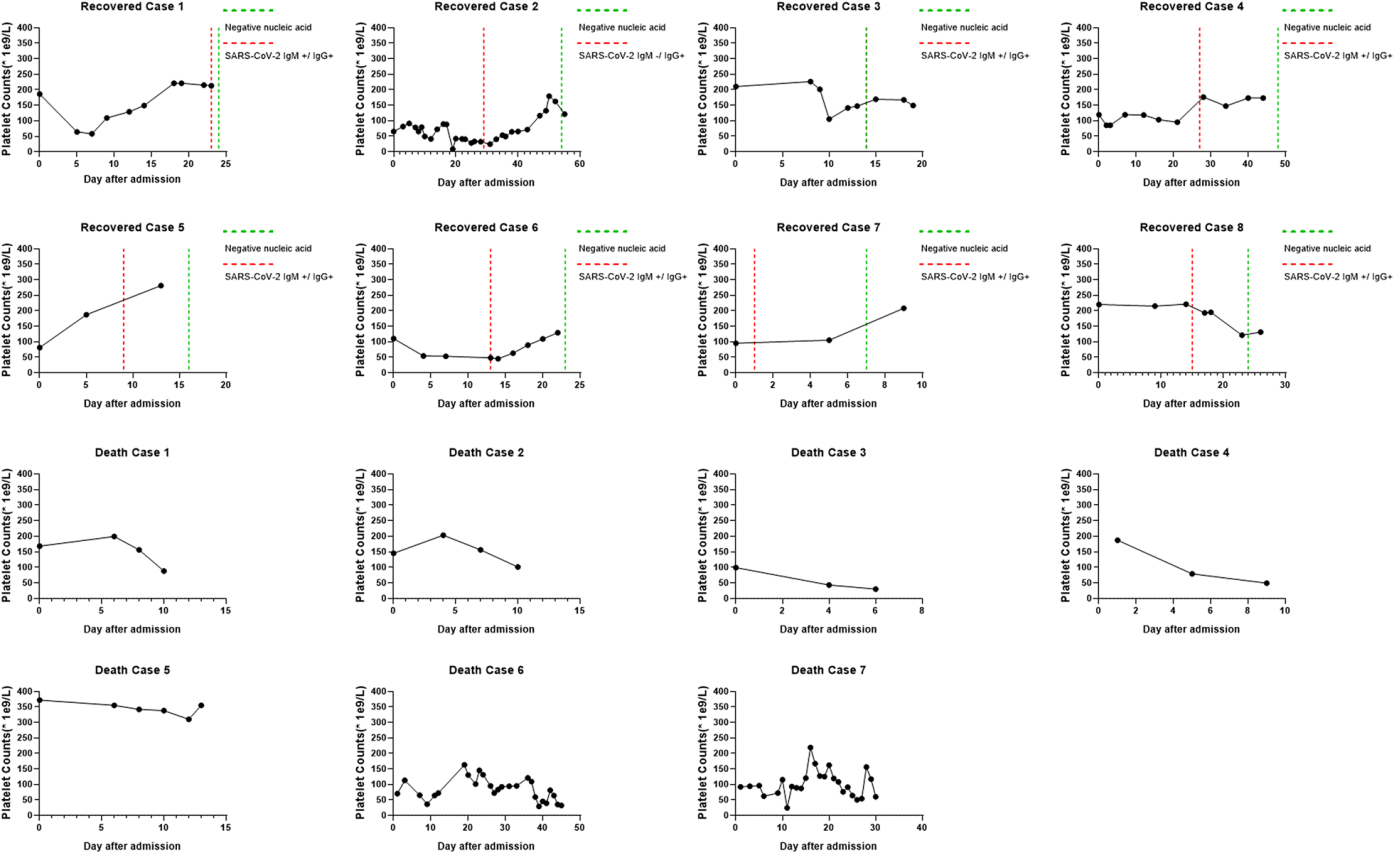
Thrombocytopenia in recovered and death cases

The death rate of all patients was 3.93% (Fig. 1), survival rate between non-severe and severe patients had significant difference (100% vs. 85.7%, *p *= 0.000). We did statistics based on the lab data at admission, we also looked at trend of platelet (Fig. 2). The severe patients showed the trend of lower platelet count, higher level of D-Dimer and higher rate of DIC at 1 week after admission. Platelet levels of sever patients were significantly lower than non-severe patients at admission, 1 and 2 weeks after admission. Both severe and non-severe patients had lowest median platelet level at 1 week after admission. In severe patients, 6 out of 7 death individuals had thrombocytopenia during hospitalization (Fig. 3), and platelet count decreased subsequently until death. In 8 recovered patients, thrombocytopenia occurred in 6 patients within 1 week after admission. Platelet levels were found to be recovered when positive SARS-CoV-2 IgM/IgG and negative coronavirus nucleic acid were found.

## Discussion

This is the first study to characterize the COVID-19 patients with dynamic changes of coagulation and platelet functions that showed biological significance and variation in non-severe and severe groups. In severe patients, platelet levels were significantly lower than non-severe patients at admission, 1 week and 2 weeks after admission [5, 6]. Severe patients also had higher white cell levels, but hemoglobin was not different between two groups at admission. Viral infection may affect hematopoiesis [7], in which SARS-CoV-2 may potentially impact megakaryocyte maturation and platelet production. Thrombocytopenia was also reported in severe acute respiratory syndrome (SARS) and Middle East respiratory syndrome (MERS) patients [8]. The mechanism behind could be abnormal megakaryocyte maturation in severe coronavirus infection [9, 10].

Thrombocytopenia is often associated with abnormal coagulation function. Severe group had 10.2% of patients with more than 3 s prolonged PT while non-severe group only had 1 patient (0.78%). Moreover, 51.1% of severe patients had D-Dimer more than 1 μg/mL vs. 15.6% of non-severe group [11]. Based on ISTH criteria for DIC diagnosis [12], there were 2 patients in severe group developed DIC at admission. Acquired coagulation defects could be further damaged by liver diseases, including evolving cirrhosis or fulminant hepatitis [13]. AST and ALT are markers for liver dysfunction, but not significant difference was observed between severe and non-severe patients. Blood glucose level was higher in severe patients, higher percentage of diabetes (51%) than non-severe group (25.6%) might be one of the explanations. Heart failure-related parameter myoglobin, inflammatory parameters CRP and LDH were much higher in severe subgroup. This suggests that the severe group had more and severe organ dysfunction and platelet coagulation activation and hyperfibrinolysis could be secondarily initiated. [14] Survival of severe disease group was much worse than non-severe group, 85.7% vs. 100%, *p *= 0.000 [15, 16].

Continuous variation of platelet and coagulation parameters were observed in severe group patients. There was a significant trend of decreased platelet count in association with a higher level of D-Dimer and a higher rate of DIC occurrence [17]. Thrombocytopenia at admission had significant correlation with coagulation function and DIC rate, while thrombocytopenia 1 week after admission had significant correlation with survival rate.

The study had limitations, due to a single center study with relatively short follow up of evaluation. However, coagulation defect and thrombocytopenia were observed in the context of disease severity and survival outcome, particularly the dynamic deterioration of platelet count and functions in the course of disease progression to expiration. In contrast, thrombocytopenia was less common in non-severe subgroup patients, and the platelet counts showed sept-wise improvement when disease showed a better control and before SARS-CoV-2 nucleic acid test became negative. It was also found that IgM and IgG SARS-CoV-2-binding antibodies were developed in association with platelet improvement and could be considered as an earlier biomarkers for disease recovery [18]. All the COVID-19 patients who had thrombocytopenia during the diagnosis but showed improved platelet counts demonstrated a better prognosis.

The underlying mechanism of thrombocytopenia and its importance to the outcome in COVID-19 patients remain enigmatic. However, dysregulated megakaryocytic maturation likely resulted from SARS-CoV-2 attack, increased platelet destruction and platelet consumption due to intravascular coagulation disturbance might play an important role [19]. Therefore, low platelet count is associated with abnormal coagulation function and increased risk of DIC, severe disease manifestation and increased mortality in patients with COVID-19.

## Conclusions

Thrombocytopenia in COVID-19 patients could be used as an effective biomarker to guide bone marrow damage, disease severity, possible deterioration of intravascular coagulation defect, and vascular endothelial activation during viral sepsis induced biological catastrophic cascades [20].

## Data Availability

All data generated or analyzed during this study are included in the manuscript.
